# Lockdown measures and relative changes in the age-specific incidence of SARS-CoV-2 in Spain

**DOI:** 10.1017/S0950268820002551

**Published:** 2020-10-21

**Authors:** P.M. De Salazar, D. Gómez-Barroso, D. Pampaka, J.M. Gil, B. Peñalver, C. Fernández-Escobar, M. Lipsitch, A. Larrauri, E. Goldstein, M.A. Hernán

**Affiliations:** 1 Center for Communicable Disease Dynamics, Department of Epidemiology, Harvard T.H. Chan School of Public Health, Boston, MA, USA; 2 Centro Nacional de Epidemiología, Instituto de Salud Carlos III, Madrid, Spain; 3 Consorcio de Investigación Biomédica en Red de Epidemiología y Salud Pública (CIBERESP), Madrid, Spain; 4 Department of Anesthesiology, Santa Creu i Sant Pau Hospital, Barcelona, Spain; 5 Department of Immunology and Infectious Diseases, Harvard T.H. Chan School of Public Health, Boston, MA, USA; 6 Department of Epidemiology and Department of Biostatistics, Harvard T.H. Chan School of Public Health; Harvard-MIT Division of Health Sciences and Technology, Boston, MA, USA

**Keywords:** Age groups, lockdown, SARS-CoV-2, Spain

## Abstract

During the first months of the severe acute respiratory syndrome-coronavirus-2 (SARS-CoV-2) epidemic in 2020, Spain implemented an initial lockdown period on 15 March followed by a strengthened lockdown period on 30 March when only essential workers continued to commute to work. However, little is known about the epidemic dynamics in different age groups during these periods.

We used the daily number of coronavirus 2019 cases (by date of symptom onset) reported to the National Epidemiological Surveillance Network among individuals aged 15–19 years through 65–69 years. For each age group *g*, we computed the proportion PrE(*g*) of individuals in age group *g* among all reported cases aged 15–69 years during the pre-lockdown period (1−10 March 2020) and the corresponding proportion PrL(*g*) during two lockdown periods (initial: 25 March−3 April; strengthened: 8–17 April 2020). For each lockdown period, we computed the proportion ratios PR(*g*) = PrL(*g*)/PrE(*g*). For each pair of age groups *g*_1_, *g*_2_, PR(*g*_1_)>PR(*g*_2_) implies a relative increase in the incidence of detected SARS-CoV-2 infection in the age group *g*_1_ compared with *g*_2_ for the lockdown period *vs.* the pre-lockdown period.

For the initial lockdown period, the highest PR values were in age groups 50–54 years (PR = 1.21; 95% CI: 1.12,1.30) and 55–59 years (PR = 1.19; 1.11,1.27). For the second lockdown period, the highest PR values were in age groups 15–19 years (PR = 1.26; 0.95,1.68) and 50–54 years (PR = 1.20; 1.09,1.31).

Our results suggest that different outbreak control measures led to different changes in the relative incidence by age group. During the initial lockdown period, when non-essential work was allowed, individuals aged 40–64 years, particularly those aged 50–59 years, had a higher relative incidence compared with the pre-lockdown period. Younger adults/older adolescents had an increased relative incidence during the later, strengthened lockdown. The role of different age groups during the epidemic should be considered when implementing future mitigation efforts.

## Introduction

The ongoing severe acute respiratory syndrome-coronavirus-2 (SARS-CoV-2) epidemic led to the implementation of mitigation strategies worldwide. To understand SARS-CoV-2 dynamics under various mitigation strategies, it is important to study the role of age because variations in transmission by age suggest differential impact of various measures such as physical distancing and workforce-related policies, which in turn has implications for epidemic control.

The rates of SARS-CoV-2 infection seem to vary by age. Serological studies found the highest seroprevalence in younger adults and older adolescents in England [1], Switzerland [2], Germany [3] and New York State outside of the greater New York City area [4]. A serological study in Spain found higher seroprevalence in older individuals than in younger ones [5].

Control measures may have differential effectiveness in different age groups [6], and the groups for which the measures are more effective may vary across populations. Social distancing seems to have been less effective among younger adults and older adolescents in Germany [7] and among persons aged 50–59 years in the Netherlands [8].

In Spain, a national lockdown was instituted on 15 March and was further strengthened to include work restrictions to non-essential workers between 30 March and 14 April [9]. This intervention had a pronounced effect on transmission that led to decreasing case counts in all regions of the country. However, much less information is available on the relative incidence of different age groups during the different stages of the lockdown. For example, age groups that were overrepresented among those who continued non-remote work between 15 and 29 March could have had an increased relative incidence during that period.

Here, we estimate changes in detected incidence of SARS-CoV-2 infection by age group after the implementation of the different lockdown measures. We applied previously developed methodology [7, 10, 11] to assess the changes in the incidence of age groups between 15 and 69 years after control measures were implemented in Spain.

## Methods

### Data sources

**Fig. 1.**
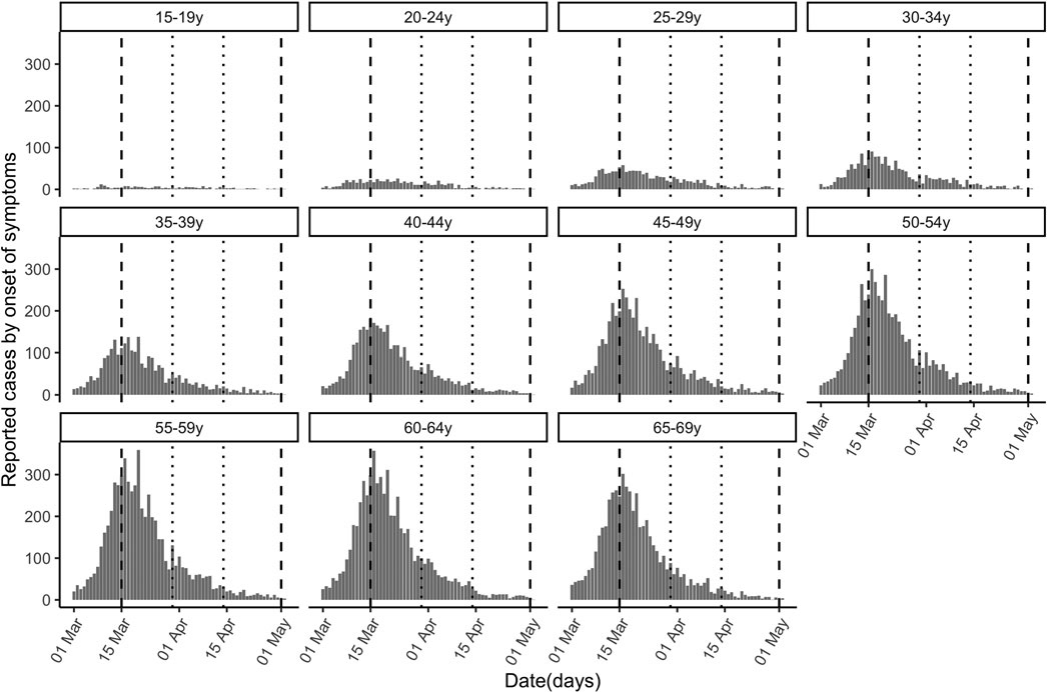
Cases of COVID-19 (reported by the day of symptom onset) by age group between 1 March and 30 April, 2020 in Spain. Vertical dashed lines demarcate the complete lockdown period (15 March−30 April) and dotted lines the strengthened lockdown period (30 March−14 April).

On 20 May we obtained information on coronavirus disease 2019 (COVID-19) cases reported from 1 March through 30 April to the National Epidemiological Surveillance Network (RENAVE) via the platform SiViEs (see Fig. 1, Results). We retrieved data on reported PCR-confirmed cases with available information on the date of symptoms onset. We excluded healthcare workers due to significant non-community transmission among them.

### Relative change in SARS-CoV-2 infection by age group

Daily counts (by date of symptom onset) of reported COVID-19 cases in individuals aged 15 through 69 years are plotted in Figure 1. We excluded children under 15 years because of potential temporal changes in diagnosis/ascertainment of cases, and individuals 70 years and older because significant non-community transmission of infection in long-term care facilities (LTCFs) likely affected their relative share of cases [12–14]. In sensitivity analyses, we restricted the calculations to (a) two regional clusters to explore heterogeneity based on seroprevalence levels and (b) hospitalised cases to evaluate the impact of potential changes in ascertainment, under the assumption that the detection of severe cases requiring hospitalisation is relatively insensitive to changes in diagnostic criteria.

We selected three periods: 1–10 March (five days before the national lockdown, as some social distancing started on 10 March), 25 March−3 April (starting ten days after implementation of the initial lockdown to detect changes in symptoms onset) and 8–17 April (starting ten days after implementation of the strengthened lockdown). During the initial lockdown, but not during the strengthened lockdown, non-essential workers were allowed to work when remote work was not possible [9].

Using the methodology in [7], we computed the age-specific proportion ratios for each of the lockdown periods (25 March−3 April or 8–17 April) relative to the pre-lockdown period (1–10 March) as follows: for each age group *g*, let *E*(*g*) be the number of COVID-19 cases in age group *g* and 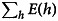 the total number of cases in age groups *h* = 1 (15–19 years) to *h* = 11 (65–69 years) during the pre-lock down period. Similarly, let *L*(*g*) and 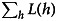 be the corresponding numbers during the initial lockdown. The proportion ratio (PR) statistic in age group *g* is
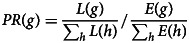
that is, the ratio between the proportion of cases in age group *g* in the initial lockdown period and the proportion of cases in age group *g* in the pre-lockdown period. We repeated these calculations after replacing the cases in the initial lockdown period by the cases in the strengthened lockdown period.

In sensitivity analyses, we computed the age-group PR statistic (a) disaggregating the cases in two groups depending on regional seroprevalence estimates [15] and (b) including only hospitalised cases, which would be less affected by potential changes in ascertainment given higher severity. The Supplementary Material describes the methods to obtain confidence bounds for the PR statistic [16].

## Results

Table 1 classifies the confirmed cases by age group and period. The higher number of cases in older individuals compared with younger ones does not necessarily reflect differences in incidence because infections are more severe in older individuals, and the likelihood of reporting of infection is higher for severe infections. Figure 1 plots the epidemic curves of daily COVID-19 cases by age group (15–69 years) between 1 March and 30 April, 2020 (*n* = 73 650).

**Table 1.** Number of reported COVID-19 cases with available information on the date of symptom onset in different age groups for different time periods

Period (years)	15–19	20–24	25–29	30–34	35–39	40–44	45–49	50–54	55–59	60–64	65–69
Pre-lockdown 1−10 March	113	250	399	482	646	795	942	970	1065	1200	1342
Initial lockdown 25 March–3 April	139	346	545	674	1004	1409	1783	2032	2200	2177	1882
Strengthened lockdown 8−17 April	76	135	215	278	330	441	506	618	657	593	522

Case counts increased a great deal with age, likely reflecting higher case ascertainment for older individuals.

### After the initial lockdown period

**Fig. 2.**
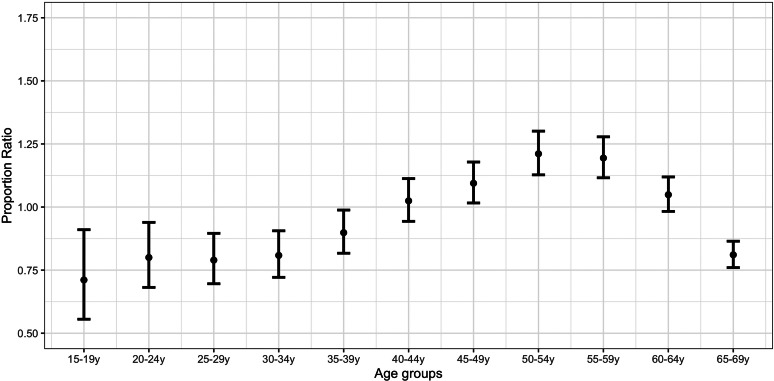
Proportion ratio estimates of confirmed COVID-19 cases by age group in Spain for the period 25 March–3 April *vs.* 1−10 March.

Figure 2 plots the PR estimates for the initial lockdown period compared with the pre-lockdown period. The highest estimates correspond to individuals aged 50–54 years (PR = 1.21; 95% CI 1.12,1.30) and 55–59 years (PR = 1.19; 1.11,1.27). PR estimates for individuals aged 15–44 years and 60–69 years were significantly lower than the estimates for those aged 50–59 years (Supplementary Material, online Supplementary Table S2).

### After the strengthened lockdown period

**Fig. 3.**
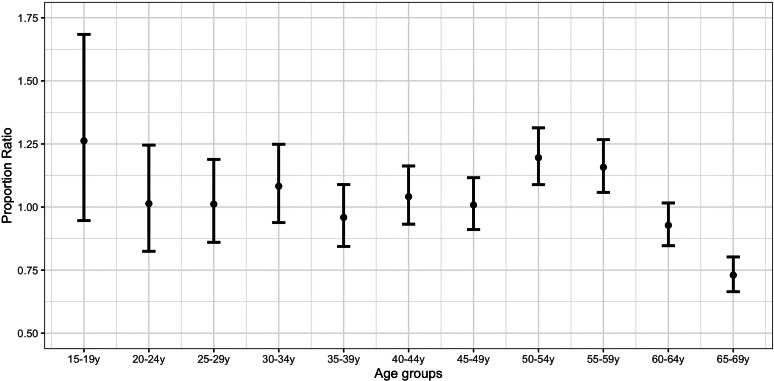
Proportion ratio estimates of confirmed COVID-19 cases by age group in Spain for the period 8−17 April *vs.* 1−10 March.

Figure 3 plots the PR estimates for the strengthened lockdown period *vs.* the pre-lockdown period. The highest estimates correspond to individuals aged 15–19 years (PR = 1.26; 0.95,1.68), 50–54 years (PR = 1.20; 1.09,1.31) and 55–59 years (PR = 1.16; 1.06,1.27). PR estimates for individuals aged 50–54 years were significantly higher than the estimates for those aged 35–49 years and 60–69 years (Supplementary Material, online Supplementary Table S3).

A comparison of Figures 2 and 3 suggests an increase in the proportion of COVID-19 cases for the strengthened lockdown period compared with the initial lockdown period in younger age groups (up to 34 years) relative to the middle ones (35–64 years).

In sensitivity analyses, PR estimates did not materially change across regions with different seroprevalence or when we restricted the analysis to hospitalised cases (Supplementary Material, Sections S1–S2).

## Discussion

We applied the methodology described in [7, 10, 11] to show that the relative incidence of detected SARS-CoV-2 infections in different age groups changed during the lockdown periods in Spain. Compared with the pre-lockdown period, individuals aged 40–64 years, and particularly those aged 50–59 years, had an elevated relative incidence during the first lockdown period. The corresponding relative incidence of younger adults/older adolescents, as well as persons aged 50–59 years, increased during the strengthened lockdown period compared with the pre-lockdown period.

These differences by age group might be explained by a number of factors. First, the elevated relative incidence in middle-aged adults during the first lockdown period, when non-essential workers were allowed to work, is consistent with the higher employment rates in Spain in middle-aged adults compared with younger adults [17]. Second, adherence to social distancing may vary with age: relative increases for younger adults/older adolescents during the second lockdown period might reflect lower adherence to lockdown measures, whereas perception for risk of severe disease could have led to stronger individual adherence for persons aged 60–69 years. Finally, household transmission in the context of the high proportion of multigenerational households in Spain may also have contributed to the observed patterns. Further work is needed to understand those issues to better inform future mitigation efforts.

Our results are aligned with findings in other European countries. Persons aged 50–59 years were least impacted in terms of mixing/number of contacts with people following the introduction of social distancing measures in the Netherlands [8], which is in agreement with the elevated relative incidence in persons (excluding healthcare workers) aged 50–59 years throughout the lockdown period in Spain. Our findings for the strengthened lockdown period in Spain echo those from other countries: a higher relative incidence of infection in younger persons was found in England [1], Switzerland [2] and Germany (Figure 6A in [3]), and the highest proportion ratio estimates for the post lockdown period in Germany are in younger persons [7].

Our findings could be affected by age-differential changes in case ascertainment, over time or across regions. However, there is no evidence of fundamental diagnostic changes for the adult population during the lockdown period in Spain, where the focus was on testing the more severe cases. Moreover, our sensitivity analysis restricted to hospitalised cases, which are less likely affected by changes in ascertainment, as well as region-specific analyses (with splitting into regions reflecting places with higher and lower-medium seroprevalence) yielded estimates that were consistent with those of the main analysis.

In summary, our paper provides evidence for an elevated relative incidence of individuals aged 40–64 years during the initial lockdown period, when non-essential work was allowed, and for an elevated relative incidence of younger adults/older adolescents when only essential workers continued to work. These results suggest that age structure is an important factor in the effect of lockdown interventions.

## Data Availability

The data that support the findings of the study are available as aggregates in Table 1 of the main text (and at the end of the supplementary material, online Supplementary Table S1 for sensitivity analysis). Line list data are available upon request from the National Center of Epidemiology, Carlos III Institute of Health, Madrid, Spain.

## Supplementary material

For supplementary material accompanying this paper visit https://doi.org/10.1017/S0950268820002551. click here to view supplementary material
