# Supplementary material for: Lockdown measures and relative changes in the age-specific incidence of SARS-CoV-2 in Spain
Source: Epidemiol Infect. 2020 Oct 21;148:e268. doi: 10.1017/S0950268820002551 (PMC7674783; doi:10.1017/S0950268820002551)
Supplement: Supplementary file 1 [file S0950268820002551sup001.docx]

Supplementary Material for “**Lockdown measures and relative changes in the age-specific incidence of SARS-CoV-2 in Spain”**

**Section S1: Proportion ratios by seroprevalence cluster**

A simple hierarchical agglomerative cluster analysis was performed using SARS-CoV-2 regional seroprevalence estimates by May 11 [[5]](https://paperpile.com/c/f1WNvc/uqyX) to compute the distance of merge (height) using Ward’s Criterion [[18]](https://paperpile.com/c/f1WNvc/J9Jj). The resulting cluster dendrogram (Figure S1) allowed us to classified Spanish regions (“comunidades autónomas”, Figure S2) in two clusters which also were geographically consistent: a central cluster (around Madrid), which had high serologically confirmed incidence rates (>9%), and a peripheral cluster with low-to-medium incidence rates (2-6%). The analysis was implemented in R (v4.0.2). Overall, no significant differences were observed regarding the computed PR with the highest estimates belonging to persons aged 50-59y for the first period and with a relative increase in old adolescents/younger adults (15-34y) in addition to those 50-59y during the second one as seen in Figure S3 A-B.


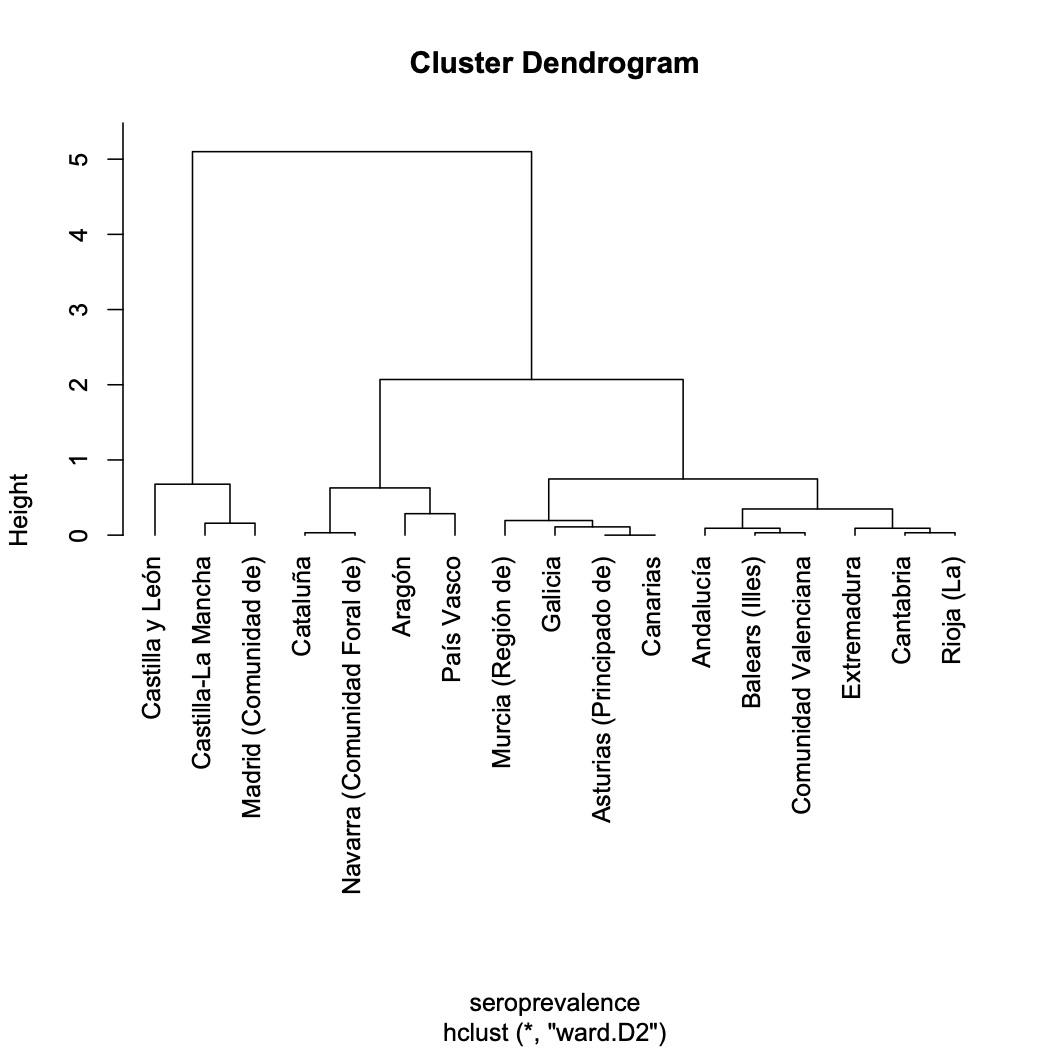

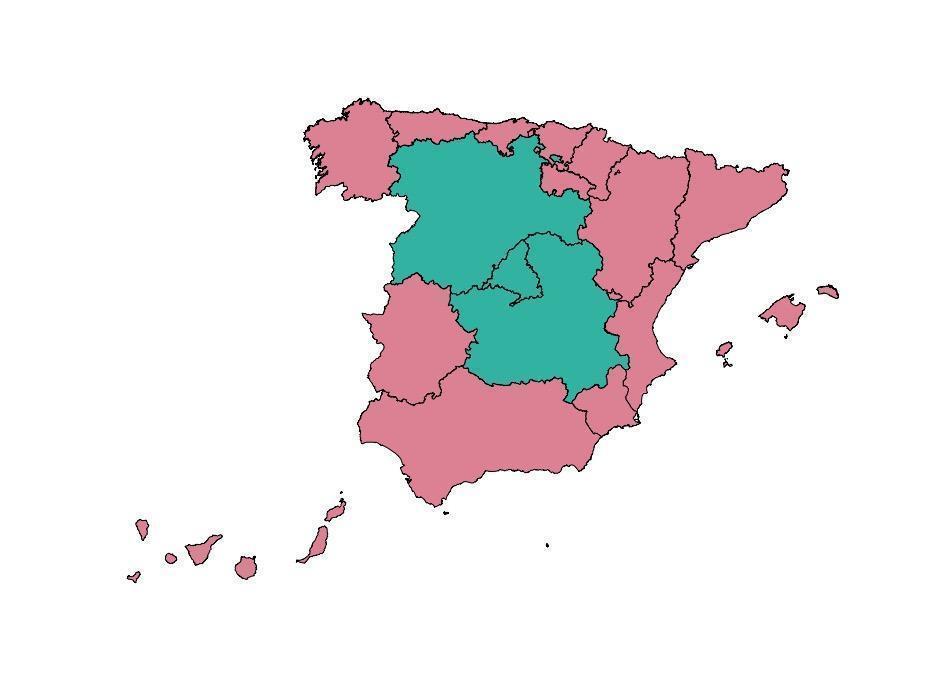


**Figure S1**: Splitting of Spain into a high seroprevalence cluster (central) and low-to-medium seroprevalence cluster (peripheral) using regional seroprevalence estimates as distance (height) variable .

***A: High seroprevalence cluster***


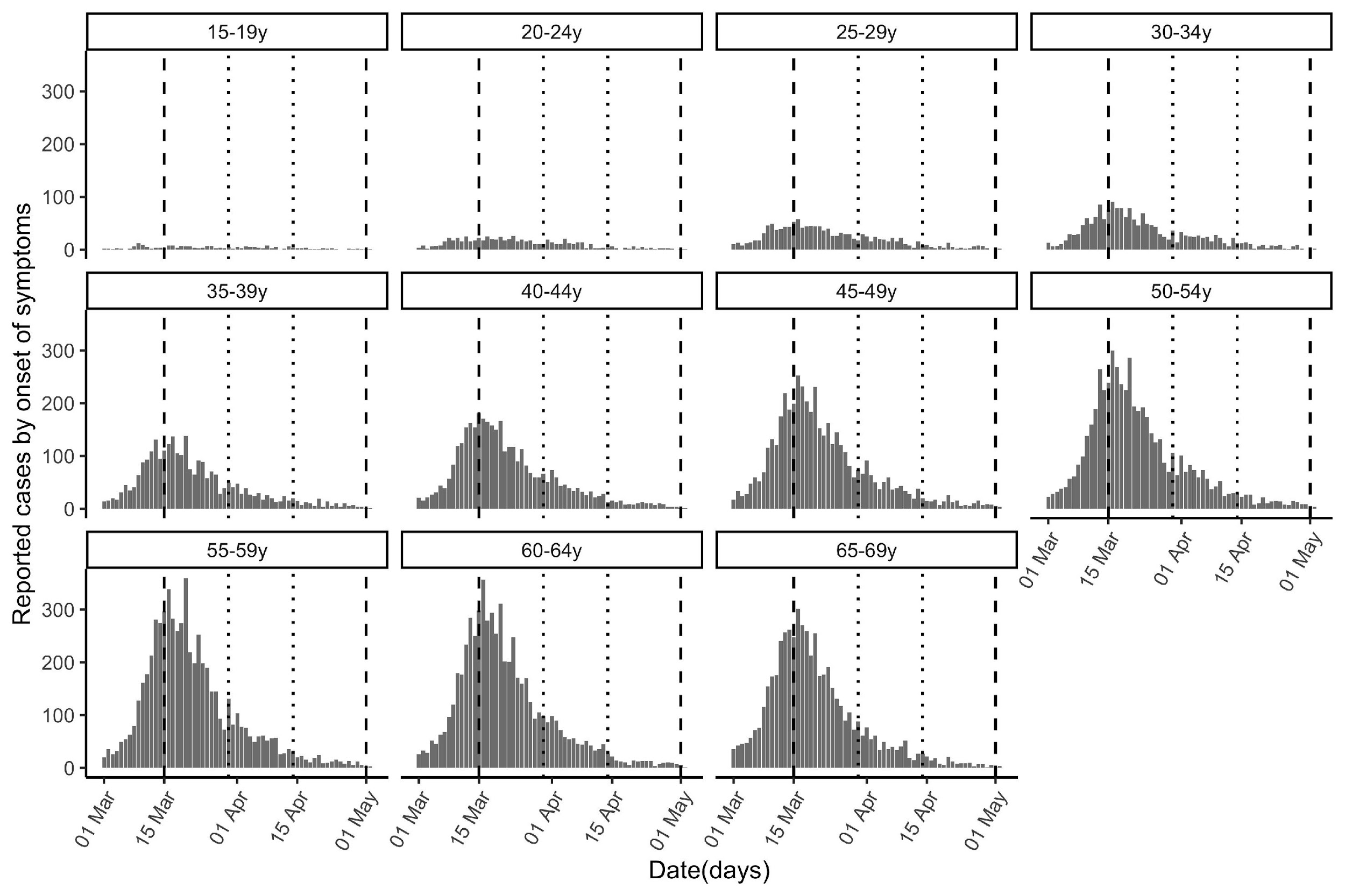


***B: Low-medium seroprevalence cluster*
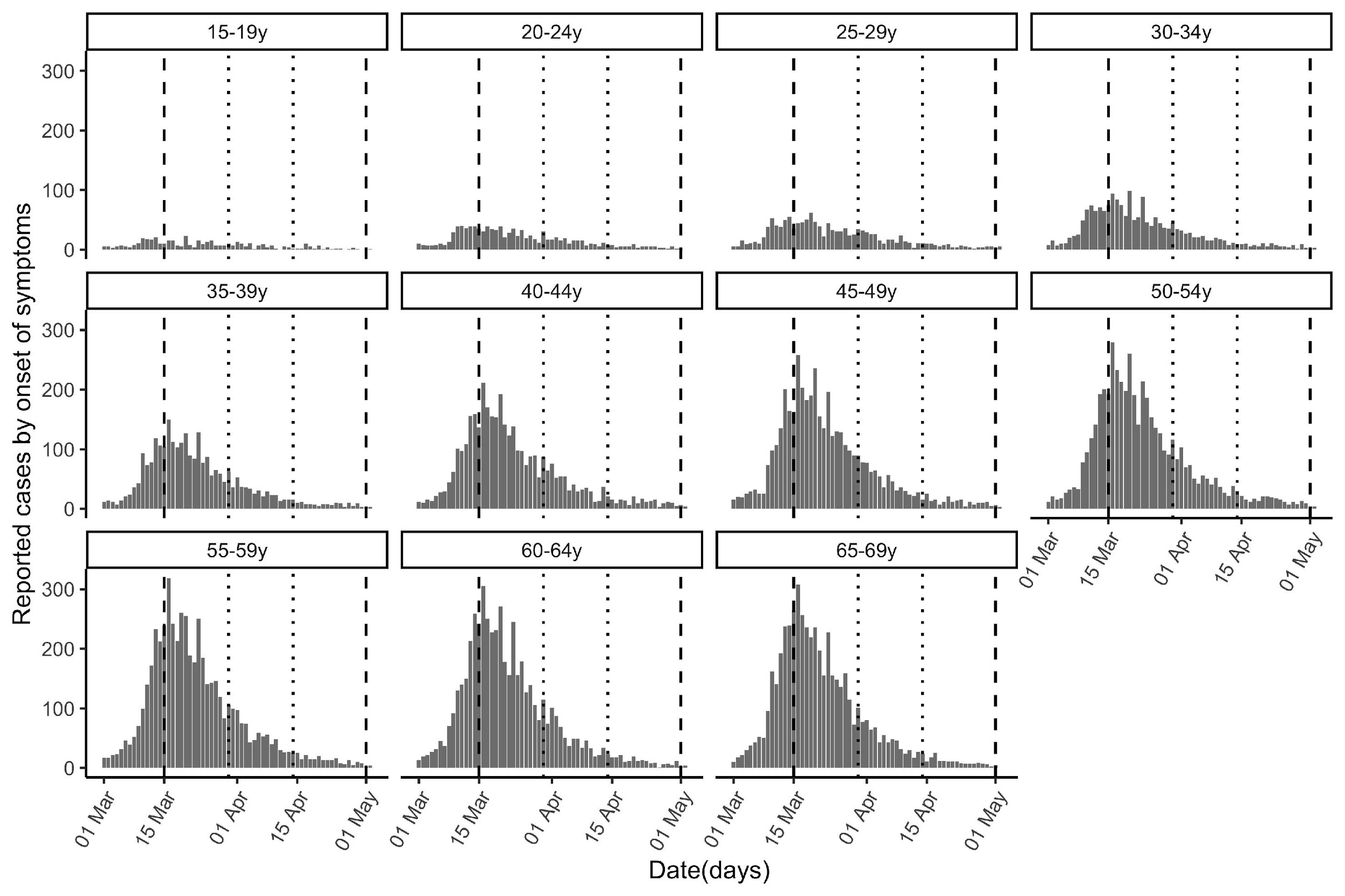
**

**Figure S2:** Cases of COVID-19 (reported by the day of symptom onset) by age group between March 1 and April 30, 2020 in each the Central cluster (A) and Peripheral cluster (B) in Spain. Vertical dashed lines demarcate the complete lockdown period (15 March to 30 April) and dotted lines the second (strengthened) lockdown period (30 March-14 April).

A.


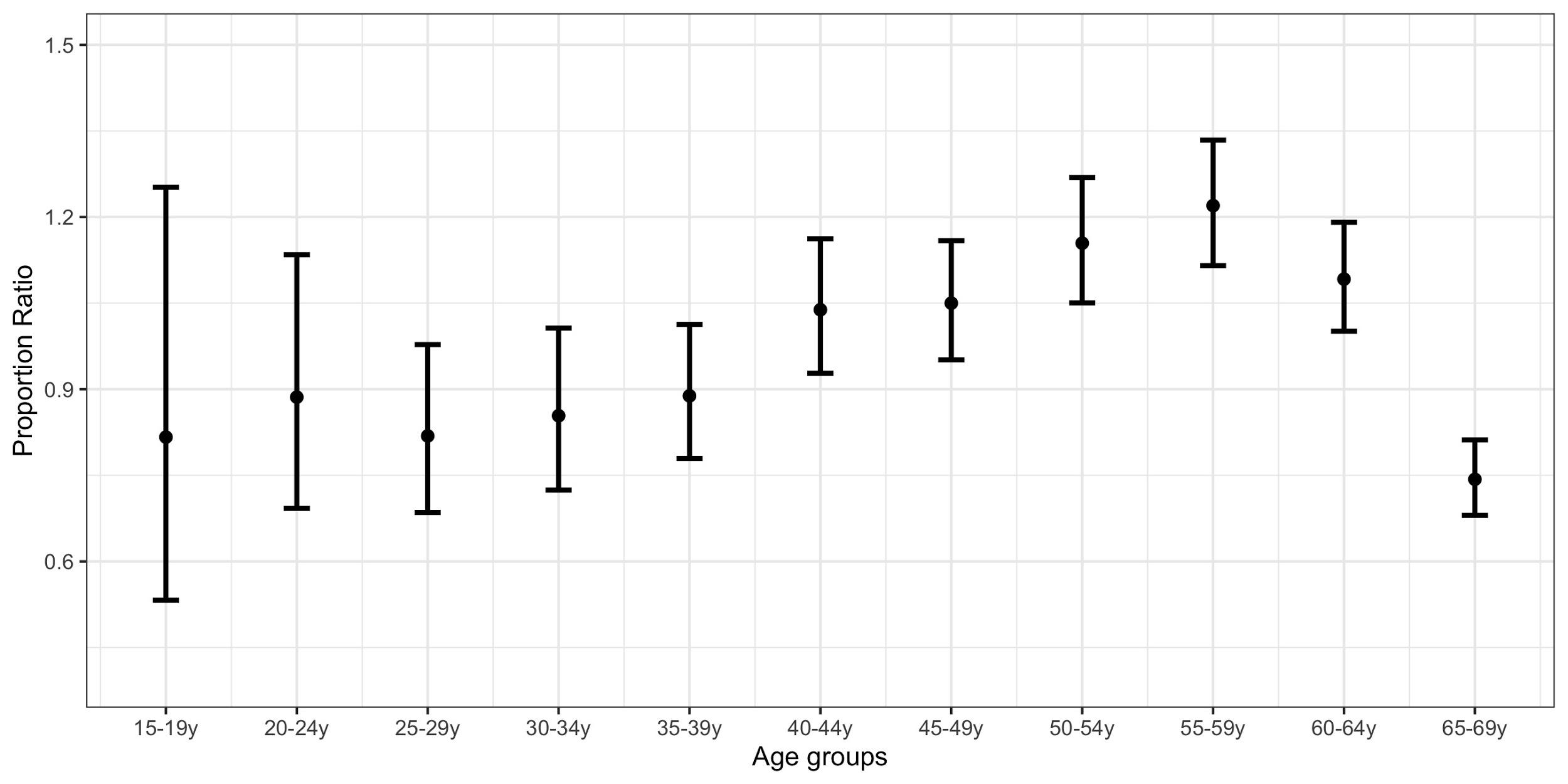


B


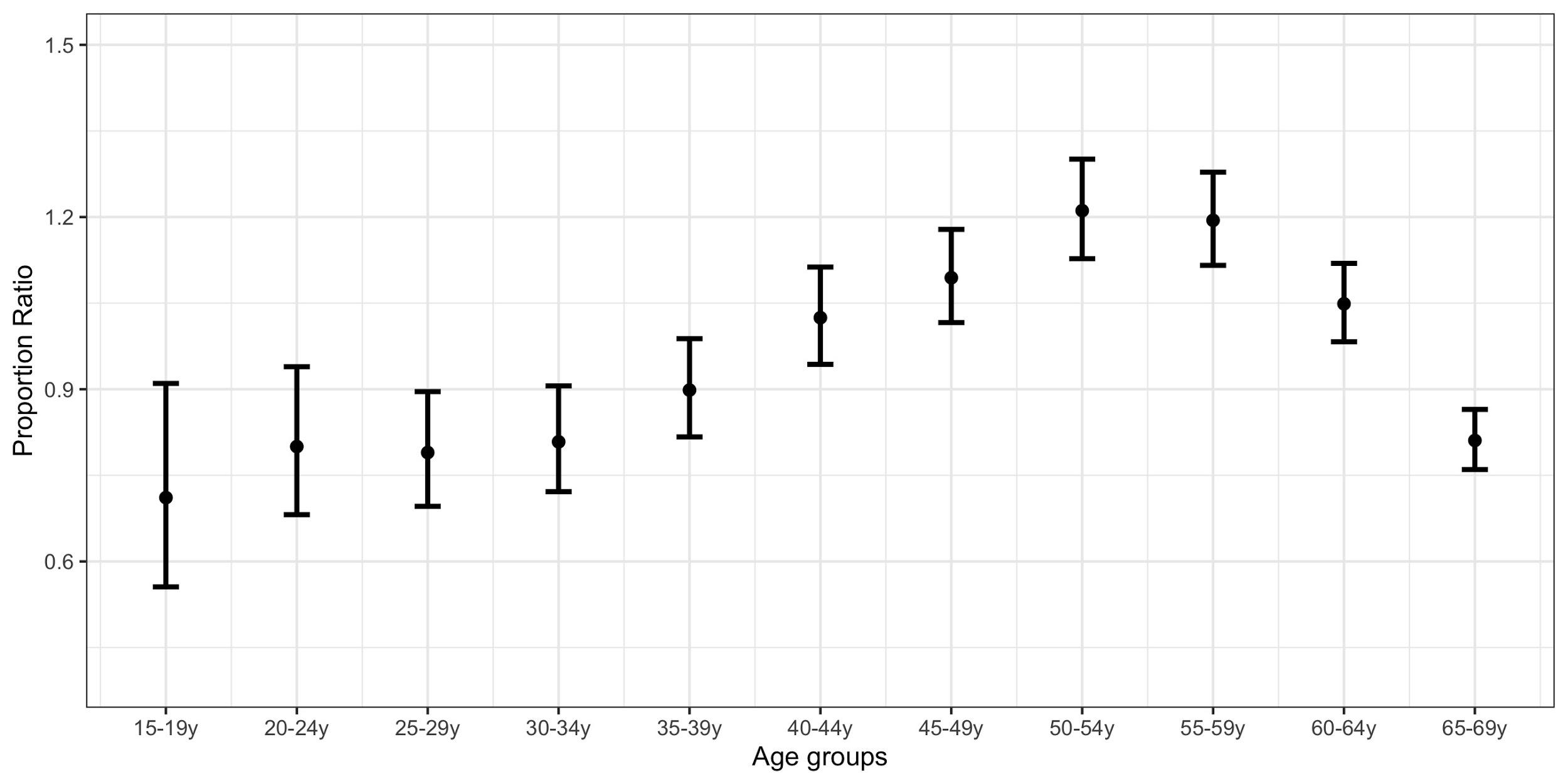


**Figure S3**: Proportion ratio estimates of confirmed COVID-19 cases by age group in Spain for the period 25 March–3 April vs. 1-10 March for the Central cluster with high seroprevalence (A) and the Peripheral cluster with low-medium seroprevalence (B).

A


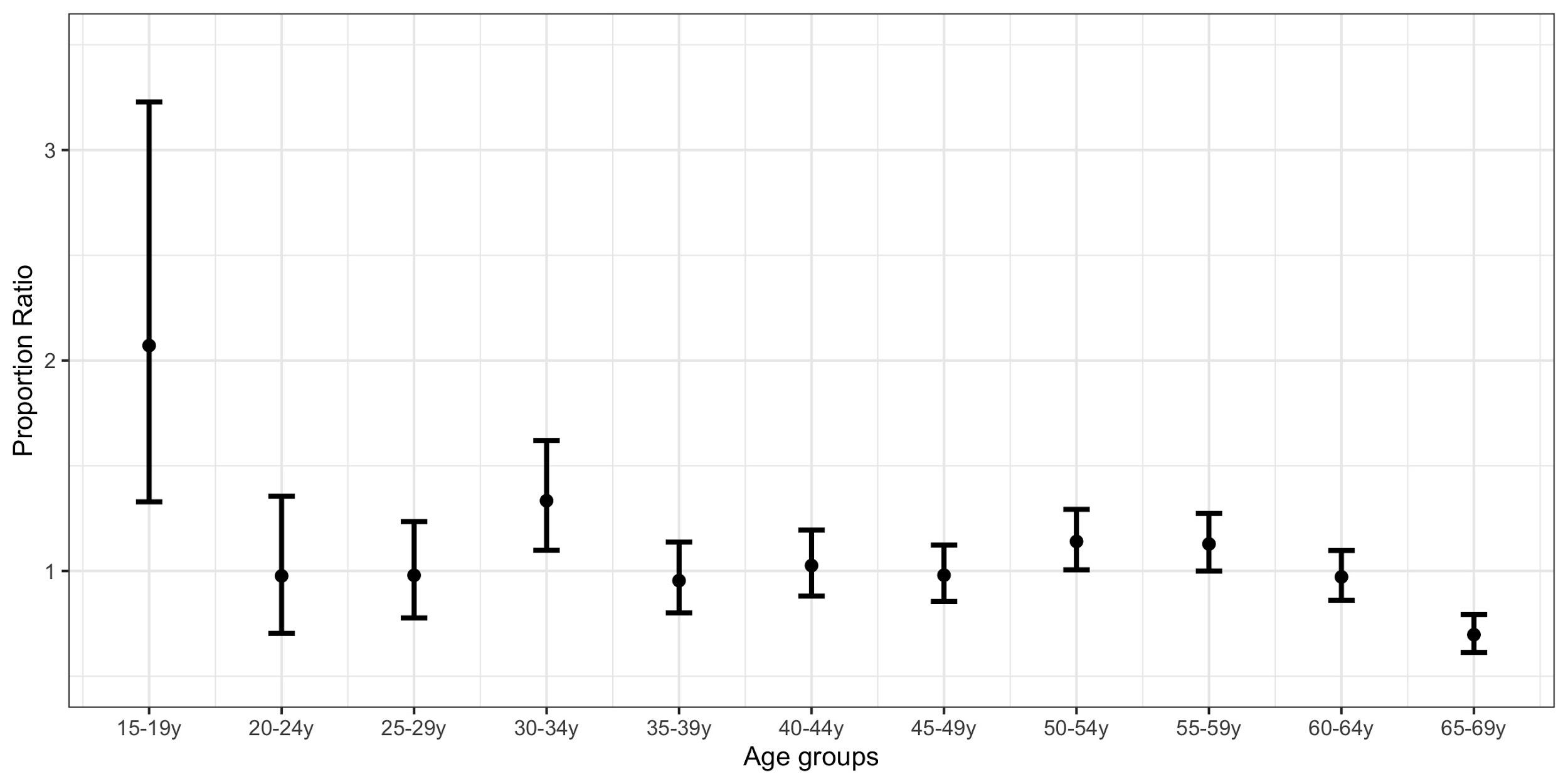


B


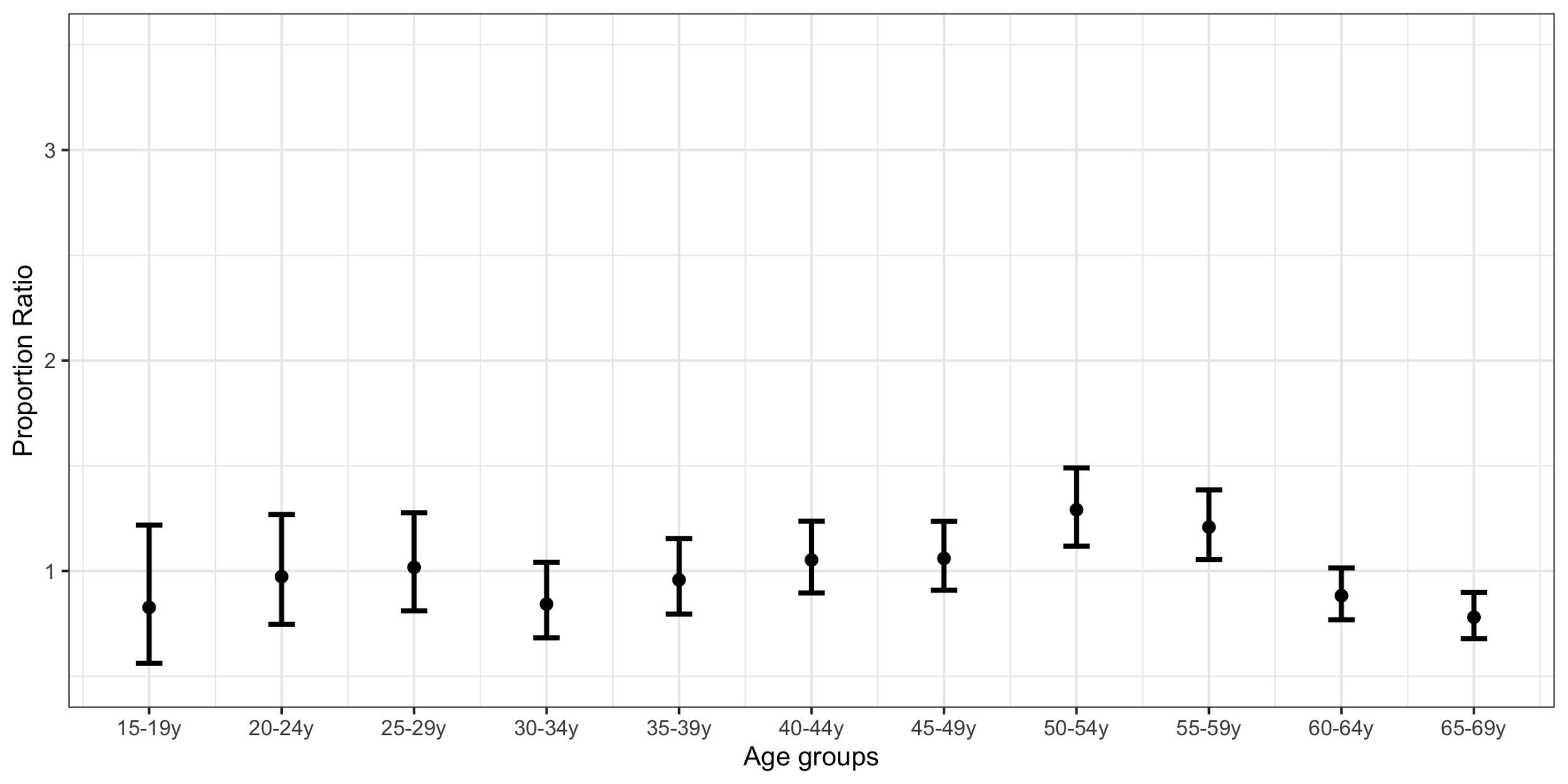


**Figure S4**: Proportion ratio estimates of confirmed COVID-19 cases by age group in Spain for the period 8-17 April vs. 1-10 March for the Central cluster with high seroprevalence (A) and the Peripheral cluster with low-medium seroprevalence (B).

**Section S2**: Proportion ratio by age-group for hospitalized cases.

Figure S5 plots the epidemic curves of daily (by the day of symptom onset) hospitalized COVID-19 cases for eleven 5-year age groups: (15-19y through 65-69y) between 1 March and 30 April, 2020 (n=37834, obtained from RENAVE /SiViEs for those with available information, see Methods). Table S1 summarizes the number of hospitalized cases reported by age group for each period used in the analysis. Figure S5A plots the estimates of the proportion ratio (PR) for the period of 25 March –3 April vs. 1 March –10 March (eq. 1) and Figure S5B plots PR for the period 8-17 April vs 1 March -10 March for individuals requiring hospitalization. While the confidence bounds in Figures S5A, B are wide due to smaller sample size, especially within younger age groups, the point estimates shown in Figure S5A are in alignment to those shown in Figure 2 with the highest PR estimates belonging to persons aged 50-59y. Similarly, the estimates shown in Figure S5B are consistent with those in Figure 3, with a relative increase in old adolescents/younger adults (15-34y) in addition to those 50-59y.


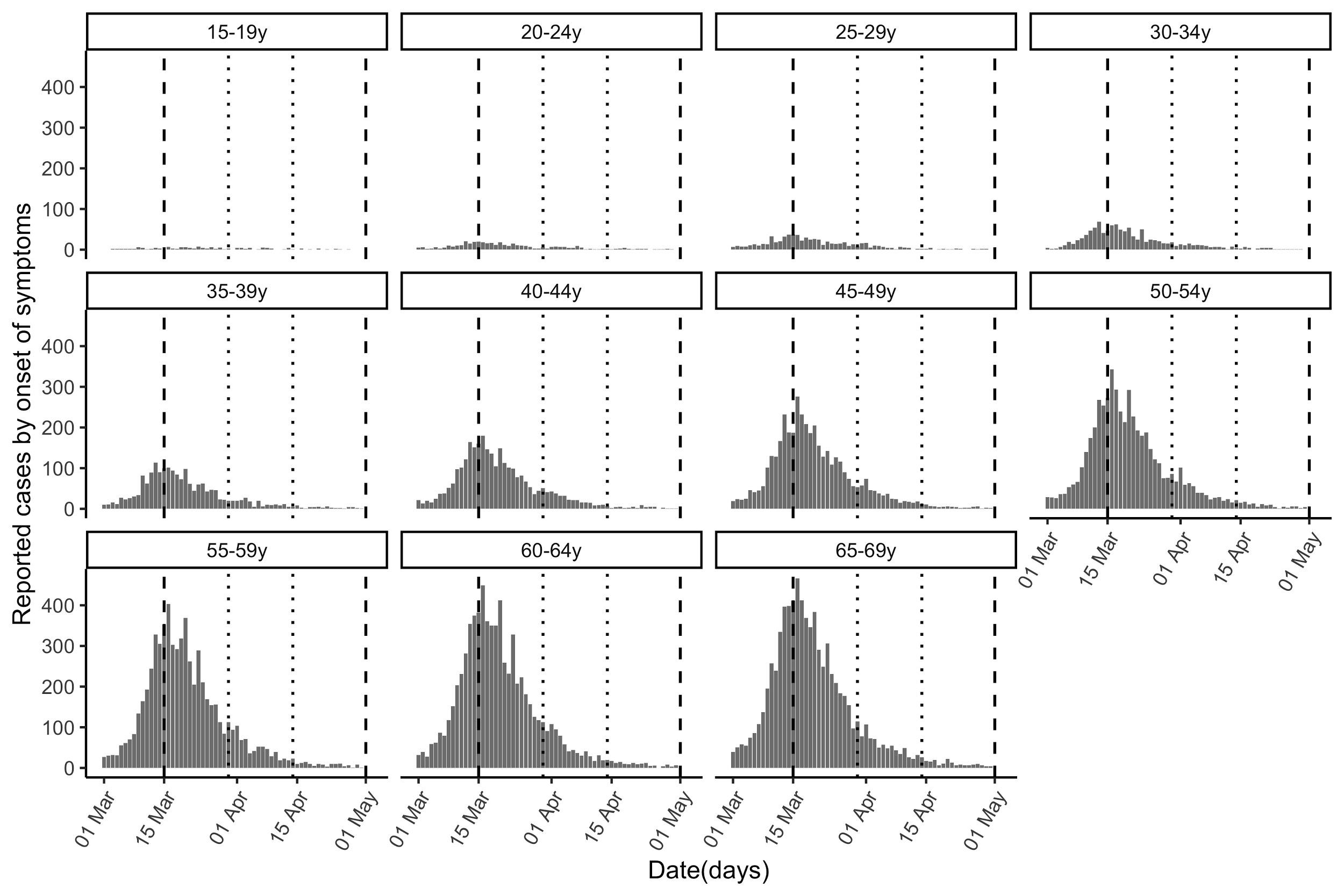


**Figure S5:** Cases of hospitalized COVID-19 cases (reported by the day of symptom onset) by age group between March 1 and April 30, 2020.

| **Period** | **15-19y** | **20-24y** | **25-29y** | **30-34y** | **35-39y** | **40-44y** | **45-49y** | **50-54y** | **55-59y** | **60-64y** | **65-69y** |
| --- | --- | --- | --- | --- | --- | --- | --- | --- | --- | --- | --- |
| Pre-lockdown  1-10 March | 22 | 58 | 126 | 147 | 271 | 381 | 509 | 577 | 688 | 859 | 1060 |
| Initial lockdown  25 March – 3 April | 30 | 63 | 125 | 166 | 289 | 487 | 737 | 908 | 1127 | 1291 | 1264 |
| Strengthened lockdown 8-17 April | 16 | 20 | 27 | 52 | 72 | 92 | 144 | 203 | 236 | 231 | 285 |

**Table S1**: Number of hospitalized COVID-19 cases with available information on the date of symptom onset in different age groups for different time periods.

A.


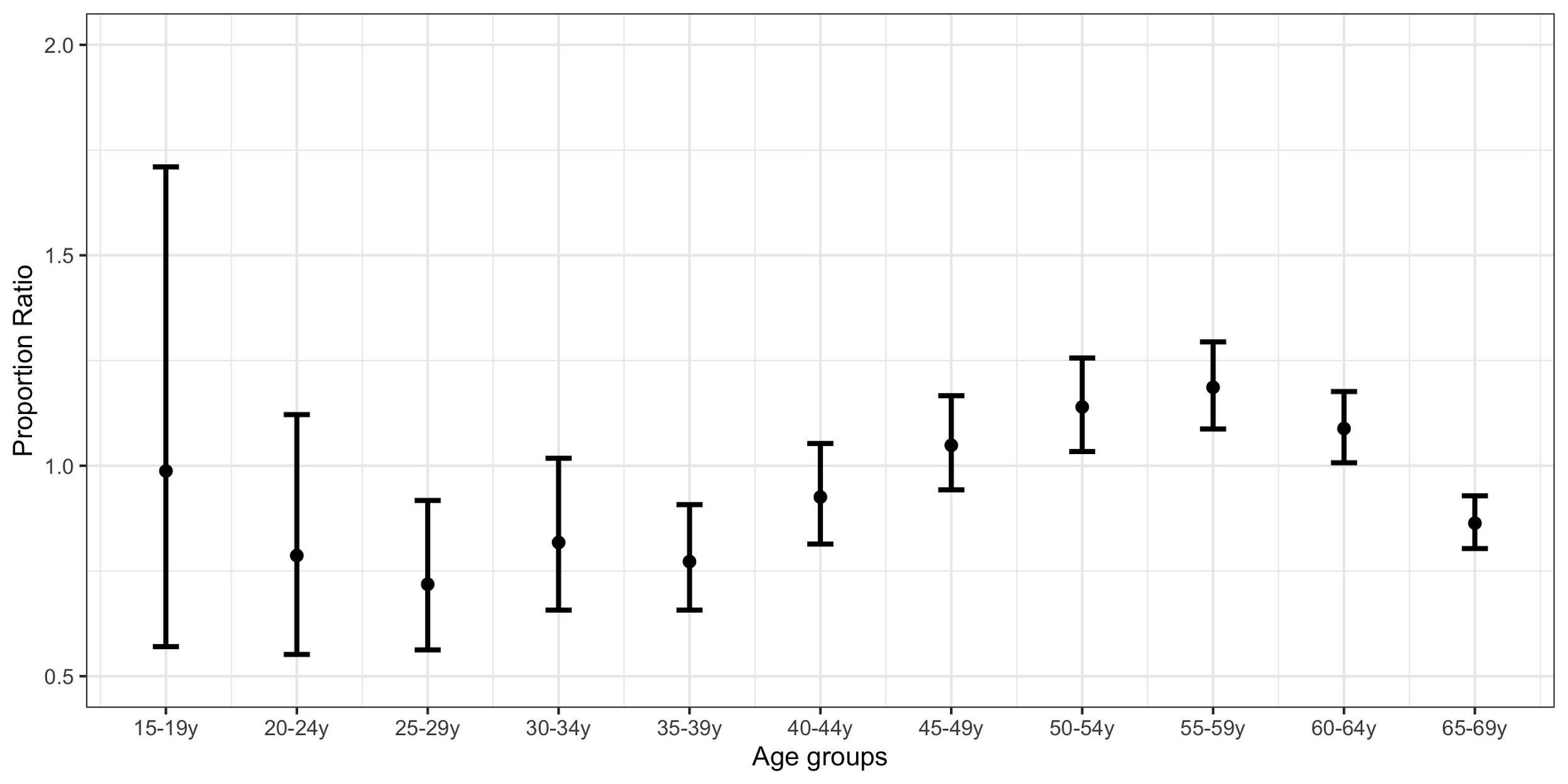


B


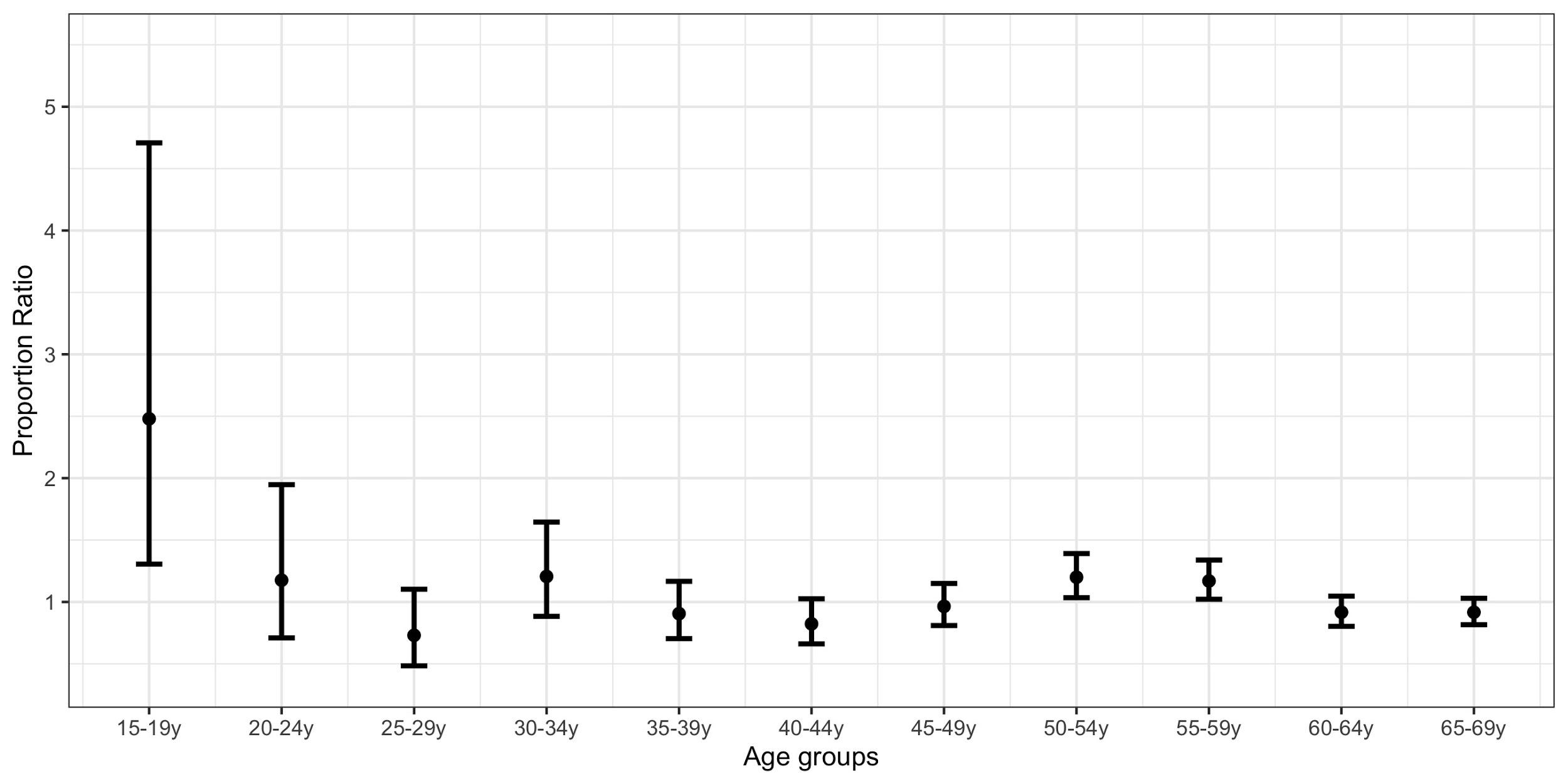


**Figure S5**: Proportion ratio estimates for hospitalized cases the period of 25 March– 3 April 3 vs. 1 March –10 March (A) and 8 April –17 April vs. 1 March –10 March 10

**Section S3:** Confidence bounds and pairwise comparison for proportion ratios

For the proportion ratio statistic introduced in the main text (eq. 1), the logarithm ln(*PR*(*g*)) of the PR(*g)* is approximately normally distributed [14] with the standard error:

$$SE=\sqrt{\frac{1}{L(g)}+\frac{1}{E(g)}-\frac{1}{\sum_{h} L\left( h \right)}-\frac{1}{\sum_{h} E(h)}}(S1)$$

For each pair of age groups *g*1 and *g*2, the proportion ratios *PR*(*g*1) and *PR*(*g*2) are compared using

the odds ratio

$$OR\left( g1,g2 \right)=\frac{PR\left( g1 \right)}{PR(g2)}$$

The odds ratios provide a pairwise comparison between *PR*(*g1*) and *PR*(*g2*), and allow one to assess whether the *PR*(*g1*) is significantly bigger (with 95% confidence) than *PR*(*g2*). It follows from eq. 1 in the main body of the text that *OR*(*g1*,*g2*) equals

$$OR=\frac{\frac{L(g1)}{E(g1)}}{\frac{L(g2)}{E(g2)}}(S2)$$

which is the OR for a COVID-19 case to be in age group *g*1 vs *g*2 for the later vs early period. Estimates for pairwise OR were performed using Fisher’s exact test. Table S2 gives the estimates of the odds ratios (ORs) for different pairs of age groups (15-29y through 65-69y) for a COVID-19 case to be detected for the period of 25 March –3 April vs. 1-10 March (eq. 3). Table S1 shows that for persons aged 50-59y, the corresponding odds ratio relative to any age group either between 15-39y, or above 60y is significantly above 1. For persons aged 45-49y, the corresponding odds ratio relative to any age group either between 15-39y, or 65-69y is significantly above 1.

| **Age group**  **(years)** | **20-24** | **25-29** | **30-34** | **35-39** | **40-44** | **45-49** | **50-54** | **55-59** | **60-64** | **65-69** |
| --- | --- | --- | --- | --- | --- | --- | --- | --- | --- | --- |
| **15-19** | 0.89  (0.65-1.21) | 0.9  (0.6-1.20) | 0.88  (0.61-1.17) | 0.79  (0.57-1.04) | **0.69**  **(0.53-0.91)** | **0.65**  **(0.5-0.85)** | **0.59**  **(0.45-0.77)** | **0.60**  **(0.46-0.78)** | **0.68**  **(0.52-0.89)** | 0.88  (0.67-1.15) |
| **20-24** |  | 1.01  (0.82-1.25) | 0.99  (0.81-1.22) | 0.89  (0.73-1.08) | **0.78**  **(0.65-0.94)** | **0.73**  **(0.61-0.88)** | **0.66**  **(0.55-0.79)** | **0.67**  **(0.56-0.80)** | **0.76**  **(0.64-0.92)** | 0.99  (0.82-1.18) |
| **25-29** |  |  | 0.98  (0.82-1.17) | 0.88  (0.74-1.04) | **0.77**  **(0.66-0.90)** | **0.72**  **(0.62-0.84)** | **0.65**  **(0.56-0.76)** | **0.66**  **(0.57-0.77)** | **0.75**  **(0.65-0.87)** | 0.97  (0.84-1.13) |
| **30-34** |  |  |  | 0.90  (0.77-1.05) | **0.79**  **(0.68-0.92)** | **0.74**  **(0.64-0.85)** | **0.67**  **(0.58-0.77)** | **0.68**  **(0.59-0.78)** | **0.77**  **(0.67-0.89)** | 1.0  (0.87-1.15) |
| **35-39** |  |  |  |  | 0.88  (0.77-1) | **0.82**  **(0.72-0.93)** | **0.74**  **(0.65-0.84)** | **0.75**  **(0.66-0.85)** | **0.86**  **(0.76-0.97)** | 1.11  (0.98-1.25) |
| **40-44** |  |  |  |  |  | 0.94  (0.83-1.05) | **0.85**  **(0.75-0.95)** | **0.86**  **(0.76-0.96)** | 0.98  (0.87-1.09) | **1.26**  **(1.13-1.42)** |
| **45-49** |  |  |  |  |  |  | 0.90  (0.81-1.01) | 0.92  (0.82-1.02) | 1.04  (0.94-1.16) | **1.35**  **(1.21-1.50)** |
| **50-54** |  |  |  |  |  |  |  | 1.03  (0.9-1.15) | **1.15**  **(1.04-1.28)** | **1.49**  **(1.34-1.66)** |
| **55-59** |  |  |  |  |  |  |  |  | **1.14**  **(1.03-1.26)** | **1.47**  **(1.33-1.63)** |
| **60-64** |  |  |  |  |  |  |  |  |  | **1.29**  **(1.17-1.43)** |

**Table S2.** ORs for different pairs of age groups (15-19y through 65-69y) for a COVID-19 case to be detected for the period of March 25 –April 3 vs. March 1st –March 10 (eq. 3).

Table S3 shows the estimates of the ORs for different pairs of age groups (15-19y through 65-69y) for a COVID-19 case to be detected for the period of 8-17 April vs. 1-10 March (eq. 3). Computed values in Table S3 suggests that for persons aged 40-59y, the corresponding odds ratio relative to persons aged 60-69y is significantly above 1.

| **Age**  **group**  **(years)** | **20-24** | **25-29** | **30-34** | **35-39** | **40-44** | **45-49** | **50-54** | **55-59** | **60-64** | **65-69** |
| --- | --- | --- | --- | --- | --- | --- | --- | --- | --- | --- |
| **15-19** | 1.27  (0.9-1.79) | 1.33  (0.97-1.83) | 1.27  (0.93-1.74) | **1.42**  **(1.05-1.92)** | 1.32  (0.98-1.77) | 1.34  (1-1.80) | 1.15  (0.86-1.55) | 1.22  (0.91-1.63) | **1.55**  **(1.15-2.07)** | **1.95**  **(1.45-2.61)** |
| **20-24** |  | 1.05  (0.81-1.35) | 1.  (0.79-1.28) | 1.12  (0.88-1.41) | 1.04  (0.83-1.30) | 1.06  (0.85-1.32) | 0.91  (0.73-1.13) | 0.96  (0.77-1.19) | 1.22  (0.98-1.51) | **1.53**  **(1.23-1.91)** |
| **25-29** |  |  | 0.96  (0.78-1.18) | 1.07  (0.87-1.30) | 0.99  (0.82-1.20) | 1.01  (0.84-1.22) | 0.87  (0.72-1.04) | 0.91  (0.76-1.10) | 1.16  (0.97-1.40) | **1.47**  **(1.22-1.76)** |
| **30-34** |  |  |  | 1.11  (0.92-1.34) | 1.03  (0.86-1.23) | 1.05  (0.88-1.25) | 0.9  (0.76-1.07) | 0.95  (0.81-1.13) | **1.21**  **(1.02-1.44)** | **1.53**  **(1.28-1.82)** |
| **35-39** |  |  |  |  | 0.93  (0.78-1.10) | 0.95  (0.8-1.11) | **0.81**  **(0.69-0.95)** | **0.86**  **(0.73-1.00)** | 1.09  (0.93-1.28) | **1.37**  **(1.17-1.61)** |
| **40-44** |  |  |  |  |  | 1.02  (0.88-1.19) | **0.88**  **(0.76-1.01)** | 0.92  (0.8-1.07) | **1.18**  **(1.02-1.36)** | **1.48**  **(1.28-1.72)** |
| **45-49** |  |  |  |  |  |  | **0.86**  **(0.75-0.99)** | 0.91  (0.79-1.04) | **1.15**  **(1.00-1.32)** | **1.45**  **(1.26-1.67)** |
| **50-54** |  |  |  |  |  |  |  | 1.05  (0.92-1.21) | **1.34**  **(1.17-1.53)** | **1.69**  **(1.48-1.94)** |
| **55-59** |  |  |  |  |  |  |  |  | **1.27**  **(1.11-1.45)** | **1.60**  **(1.40-1.83)** |
| **60-64** |  |  |  |  |  |  |  |  |  | **1.26**  **(1.10-1.44)** |

**Table S3.** ORs for different pairs of age groups (15-19y through 65-69y) for a COVID-19 case to be detected for the period of 8-17 April vs. 1-10 March (eq. 3).

Table S4 shows the estimates of the ORs for different pairs of age groups (15-19y through 65-69y) for a COVID-19 case to be detected for the period of 8-17 April vs. 25 March -3 April (eq. 3). Table S3 shows an increase in the incidence of detected SARS-CoV-2 infection for younger persons (aged under 34y) compared to older persons (aged over 40y) for a number of pairs of age groups.

| **Age group**  **(years)** | **20-24** | **25-29** | **30-34** | **35-39** | **40-44** | **45-49** | **50-54** | **55-59** | **60-64** | **65-69** |
| --- | --- | --- | --- | --- | --- | --- | --- | --- | --- | --- |
| **15-19** | 1.4  (0.98-2) | 1.39  (0.99-1.93) | 1.33  (0.96-1.83) | **1.66**  **(1.21-2.28)** | **1.75**  **(1.28-2.38)** | **1.93**  **(1.41-2.61)** | **1.8**  **(1.32-2.43)** | **1.83**  **(1.35-2.47)** | **2.01**  **(1.47-2.72)** | **1.99**  **(1.45-2.67)** |
| **20-24** |  | 0.99  (0.76-1.28) | 0.95  (0.74-1.21) | 1.25  (0.93-1.51) | 1.25  (0.99-1.57) | **1.37**  **(1.09-1.72)** | **1.28**  **(1.02-1.60)** | **1.31**  **(1.04-1.63)** | **1.43**  **(1.14-1.79)** | **1.41**  **(1.12-1.76)** |
| **25-29** |  |  | 0.96  (077-1.19) | 1.20  (0.98-1.47) | **1.26**  **(1.04-1.53)** | **1.39**  **(1.15-1.68)** | **1.30**  **(1.08-1.56)** | **1.32**  **(1.1-1.59)** | **1.45**  **(1.2-1.74)** | **1.42**  **(1.18-1.72)** |
| **30-34** |  |  |  | **1.25**  **(1.04.52)** | **1.32**  **(1.1-1.58)** | **1.45**  **(1.22-1.73)** | **1.36**  **(1.14-1.61)** | **1.38**  **(1.17-1.63)** | **1.51**  **(1.28-1.79)** | **1.49**  **(1.25-1.77)** |
| **35-39** |  |  |  |  | 1.05  (0.89-1.24) | 1.16  (0.98-1.36) | 1.08  (0.92-1.26) | 1.10  (0.94-1.28) | **1.21**  **(1.03-1.41)** | **1.18**  **(1.01-1.39)** |
| **40-44** |  |  |  |  |  | 1.10  (0.95-1.28) | 1.03  (0.89-1.19) | 1.05  (0.91-1.21) | **1.15**  **(1-1.33)** | 1.13  (0.97-1.31) |
| **45-49** |  |  |  |  |  |  | 0.93  (0.81-1.07) | 0.95  (0.83-1.09) | 1.04  (0.91-1.19) | 1.02  (0.89-1.18) |
| **50-54** |  |  |  |  |  |  |  | 1.02  (0.9-1.16) | 1.12  (0.98-1.27) | 1.10  (0.96-1.25) |
| **55-59** |  |  |  |  |  |  |  |  | 1.10  (0.96-1.25) | 1.08  (0.94-1.23) |
| **60-64** |  |  |  |  |  |  |  |  |  | 0.98  (0.86-1.12) |

**Table S4.** ORs for different pairs of age groups (15-19y through 65-69y) for a COVID-19 case to be detected for the period of 8-17 April vs. 25 March – 3 April (eq. 3).
